# Bridging language and culture: The LGBTQ Caregiver Acceptance Scale's Spanish (LCAS‐S) translation and validation among Latinx caregivers

**DOI:** 10.1111/jora.70254

**Published:** 2026-08-19

**Authors:** Aldo M. Barrita, Roberto L. Abreu, Alexa Parra, Cristalís Capielo Rosario, Kirsten A. Gonzalez, Melanie Domenech Rodriguez, Karina A. Gattamorta

**Affiliations:** ^1^ Department of Psychology Michigan State University East Lansing Michigan USA; ^2^ Department of Psychology University of Florida Gainesville Florida USA; ^3^ Nursing Administration Nicklaus Children's Hospital Miami Florida USA; ^4^ School of Counseling and Counseling Psychology Arizona State University Tempe Arizona USA; ^5^ Department of Psychology and Neuroscience University of Tennessee, Knoxville Knoxville Tennessee USA; ^6^ Department of Psychology Utah State University Logan Utah USA; ^7^ School of Nursing and Health Studies University of Miami Coral Gables Florida USA

**Keywords:** caregiver acceptance, Latinx LGBTQ+ youth, scale adaptation

## Abstract

Although family acceptance varies across cultural contexts, it consistently predicts wellness among LGBTQ+ people. We validated a Spanish‐language adaptation of the LGBTQ+ Caregiver Acceptance Scale (LCAS‐S) to assess culturally grounded expressions of acceptance and rejection among Spanish‐speaking Latinx caregivers of LGBTQ+ youth. Following procedures from the LCAS development study, the 40‐item measure was translated and back‐translated using established guidelines to ensure linguistic and conceptual equivalence. A total of 256 Spanish‐speaking Latinx caregivers participated, representing 15 countries of origin; the largest groups were born in the United States (46%), Mexico (20%), Puerto Rico (12%), and Cuba (6%). Scale validation included internal consistency reliability, exploratory and confirmatory factor analyses, and convergent validity testing with established measures of caregiver acceptance, family functioning, and homonegativity. The LCAS‐S demonstrated strong overall reliability (*α* = .89) and yielded a six‐factor structure: Concealment and Respeto, Supportive Actions, Public Opinion, Fear of Family Rejection, Affirming the LGBTQ+ Child's Place in the Family, and Outness. The factor structure both overlapped with and diverged meaningfully from the English version. Convergent validity patterns aligned with theoretical expectations, with restrictive dimensions correlating positively with family conflict and homonegativity and affirming dimensions correlating positively with acceptance‐related constructs. Findings provide preliminary empirical support for the LCAS‐S as a culturally and linguistically responsive tool for researchers and practitioners seeking to understand and promote acceptance among Spanish‐speaking Latinx caregivers of LGBTQ+ youth in the United States.

## INTRODUCTION

Family acceptance is a central developmental context for lesbian, gay, bisexual, transgender, and queer (LGBTQ+) adolescents and young adults (Ancín‐Nicolás et al., 2024; DelFerro et al., 2024; Ryan et al., 2010). Among Latinx LGBTQ+ youth, caregiver acceptance occurs within cultural, linguistic, and migration‐related contexts that can shape mental health and well‐being (Abreu, Barrita, et al., 2025; Abreu et al., 2026; Barrita, Abreu, et al., 2025; Barrita, Abreu, Carbajal, et al., 2026; Barrita, Abreu, Zelaya, et al., 2026; Martin et al., 2025; Ryan et al., 2009). Grounded in Interpersonal Acceptance‐Rejection Theory (IPARTheory; Rohner, 2021), caregiver acceptance and rejection reflect a universal “warmth dimension” of interpersonal relationships, ranging from expressions of love, care, and support to experiences of hostility, neglect, or emotional withdrawal. Importantly, IPARTheory emphasizes that individuals' perceptions of acceptance and rejection within close relationships are most consequential for development, highlighting caregivers as primary attachment figures whose responses shape youths' psychological adjustment across the lifespan. Although this study focuses on Latinx caregivers rather than youth directly, caregiver responses to an LGBTQ+ youth's identity represent a critical developmental context during adolescence.

Adolescence is a period characterized by identity exploration, heightened sensitivity to social evaluation, and ongoing emotional and, often, economic dependence on caregivers (Russell & Fish, 2019). For many LGBTQ+ youth, adolescence is also the developmental period during which awareness, exploration, and disclosure of sexual orientation and gender identity become increasingly salient, making caregiver responses especially consequential (Russell & Fish, 2019). For LGBTQ+ youth, including Latinx youth, these developmental processes may be further shaped by the timing of identity awareness and disclosure (Abreu et al., 2020; Russell & Fish, 2019). Contemporary developmental scholarship suggests that many youth are coming out at earlier ages, often during adolescence, when they remain embedded within family systems, making caregiver responses especially influential for youths' sense of belonging and well‐being (Russell & Fish, 2019). Accordingly, examining caregiver acceptance during adolescence provides an important lens for understanding the relational environments that support or hinder positive development among LGBTQ+ youth, particularly within Latinx families.

Research consistently suggests that Latinx LGBTQ+ youth who experience family rejection are at increased risk for depression, substance use, suicidal ideation, and other adverse outcomes (Abreu et al., 2020, 2026; Abreu, Gattamorta, et al., 2023; Ancín‐Nicolás et al., 2024; Barrita, Abreu, et al., 2025; Barrita, Abreu, Zelaya, et al., 2026; Barrita, Parmenter, et al., 2025; Ryan et al., 2009). In contrast, those who experience family acceptance report higher self‐esteem, better general health outcomes, and stronger social support networks (Abreu, Lefevor, et al., 2023; Abreu, Barrita, et al., 2025; Ancín‐Nicolás et al., 2024; Vazquez et al., 2025). For example, Ryan et al. (2009) found that Latinx LGBTQ+ youth whose families rejected them showed higher negative mental health outcomes compared to peers with accepting families. In this study, we focus specifically on Latinx LGBTQ+‐related caregiver acceptance (caregivers' attitudes, beliefs, and behaviors toward their child's sexual orientation, gender identity, and gender expression), rather than general parental warmth alone. Despite the well‐established importance of caregiver acceptance for LGBTQ+ youth development, most research and measurement tools have been developed using English‐speaking samples, limiting our understanding of how these processes operate among Spanish‐speaking Latinx families in the United States, a large and diverse sociocultural population (Pew Research Center, 2022). This limitation is particularly consequential given that language is not only a medium of communication but also a carrier of cultural values and relational norms that shape how acceptance and rejection are expressed and interpreted. Additionally, 83% of Spanish‐speaking Latinxs in the United States report that access to language‐concordant health‐related services and materials is important (Gonzalez et al., 2025), underscoring the need for culturally and linguistically responsive measurement tools. Latinx families navigate cultural values (e.g., religion and spirituality, gender norms), intergenerational traditions, and migration‐related stress that uniquely shape how caregivers respond to their LGBTQ+ child (Abreu et al., 2020; Abreu, Martin, & Badio, 2023). These cultural dimensions can both support and challenge acceptance, highlighting the need for measurement tools that are linguistically and culturally appropriate. For example, values such as familismo may shape how acceptance is expressed within family and community contexts (Calzada et al., 2010; Halgunseth et al., 2006). Research also suggests that variation in language use and acculturation may influence how caregiver acceptance is expressed and experienced (Ramos et al., 2023). As a result, examining caregiver acceptance exclusively in English may overlook culturally specific expressions of support, protection, or restriction that are more salient in Spanish‐speaking contexts.

The LGBTQ+ Caregiver Acceptance Scale (LCAS; Abreu, Gattamorta, et al., 2023) is the only known measure developed explicitly to assess culturally grounded acceptance and rejection among Latinx caregivers (i.e., the primary adult responsible for the youth) of LGBTQ+ individuals in the United States; however, it was developed and validated in English. This limitation constrains the ability to capture culturally and linguistically nuanced expressions of acceptance among Spanish‐speaking caregivers, who may conceptualize and express these processes differently. In this paper, caregiver refers to the primary adult responsible for supporting or raising the LGBTQ+ youth, whereas family refers more broadly to the larger relational or household system in which caregiver acceptance processes occur.

Language can shape attitudes and values, and the survey's language can affect data quality (Wenz et al., 2021). Within Latinx families, Spanish often serves as a key context for communicating relational expectations, particularly in immigrant and bilingual households. Because acceptance and rejection are expressed through culturally meaningful behaviors and symbols (Rohner, 2021), direct translation of measures without cultural adaptation may fail to capture the intended constructs. Without culturally and linguistically appropriate tools, scholars cannot fully examine how acceptance and rejection may differ across language contexts. Language accessibility is foundational to equity in measurement (Brown, 2015). English‐only measures risk obscuring the distinct cultural experiences of Spanish‐speaking caregivers. Thus, translating and validating scales such as the LCAS (Abreu, Gattamorta, et al., 2023) is not simply a linguistic task but a culturally appropriate mechanism for documenting diverse forms of Latinx parenting.

## LATINX LGBTQ+ CAREGIVER ACCEPTANCE AND THE LCAS

Caregiver acceptance of an LGBTQ+ child is a multidimensional process that reflects both general parenting practices and responses specific to the child's sexual orientation, gender identity, and gender expression (Ryan et al., 2010). Drawing from IPARTheory (Rohner, 2021), these processes exist along a continuum of warmth and rejection but are expressed in culturally specific ways within LGBTQ+ family contexts, particularly among Latinx families (Abreu et al., 2020; Abreu, Gattamorta, et al., 2023; Abreu, Martin, & Badio, 2023). For example, Latinx caregivers may simultaneously express love and support for their child while also encouraging concealment or limiting disclosure due to concerns about stigma and cultural values, including *respeto* [respect] (Abreu, Martin, & Badio, 2023; Halgunseth et al., 2006). This complexity highlights the need to conceptualize caregiver acceptance within Latinx youth as a culturally embedded and developmentally situated process rather than a unidimensional construct. Within Latinx families, cultural values shape how acceptance is expressed and negotiated (Abreu, Gattamorta, et al., 2023; García Coll et al., 1996; Halgunseth et al., 2006). Such values may support affirming behaviors while also contributing to restrictive responses, particularly as adolescents increasingly explore, disclose, and negotiate their LGBTQ+ identities within family and community contexts (Abreu, Lefevor, et al., 2023; Calzada et al., 2010).

The LCAS (Abreu, Gattamorta, et al., 2023) was the first validated instrument created specifically to assess culturally grounded acceptance and rejection among Latinx caregivers of LGBTQ+ youth. Rather than modifying generic parenting or family acceptance scales, the LCAS was intentionally developed with a focus on Latinx cultural values and sociocultural contexts relevant to sexual and gender minority youth. Scale construction included literature review, qualitative research, expert consultation, and adaptation of general parenting measures (e.g., Parental Acceptance‐Rejection Questionnaire [PARQ]; Rohner & Ali, 2020). The validation sample consisted of 215 English‐speaking Latinx caregivers of LGBTQ+ children across diverse sociodemographic backgrounds, including variation in national origin, religion, gender identity, and caregiver roles (e.g., biological parents, stepparents, grandparents, aunts/uncles). Factor analysis supported a 40‐item, six‐dimensional structure: Outness, Caregiver Acceptance, Concealment, Respeto, Attitudes Toward Queer Parenting, and Supportive Actions (Abreu, Gattamorta, et al., 2023). These dimensions reflect both affirming and restrictive processes, underscoring that caregiver acceptance is not simply the presence or absence of support, but a dynamic set of attitudes and behaviors that may coexist and evolve over time. This multidimensionality is especially important during adolescence, when caregivers and youth are navigating disclosure, autonomy, and belonging (Russell & Fish, 2019). One key theoretical contribution of the LCAS (Abreu, Gattamorta, et al., 2023) was demonstrating that Latinx caregivers may engage in both affirming and restrictive practices simultaneously. For example, a caregiver may express emotional support and unconditional love while also encouraging their child to conceal their identity in certain settings to avoid discrimination or social stigma. These patterns reflect efforts to balance protection, cultural expectations, and social realities. Despite these contributions, the LCAS was developed in English, limiting its ability to capture culturally and linguistically nuanced expressions of acceptance among Spanish‐speaking caregivers. Because language shapes how cultural meanings are expressed, the structure and meaning of caregiver acceptance may differ when assessed in Spanish. For example, concepts such as respeto or concerns about “qué dirán” (what others will say) may carry distinct meanings that are not fully captured through direct translation. Thus, validating a Spanish‐language version of the LCAS (LCAS‐S) is necessary to more accurately assess caregiver acceptance among Spanish‐speaking Latinx families and to understand how cultural, linguistic, and developmental contexts shape these processes. This work advances culturally grounded measurement and supports more inclusive research and intervention efforts for Latinx families and LGBTQ+ youth.

### Theoretical rationale for validating the LCAS in Spanish

Translating and validating the LCAS into Spanish provides a pathway to empirically examine how LGBTQ+‐specific caregiver acceptance and rejection operate among Spanish‐speaking caregivers. Building on developmental ecological frameworks, youth development unfolds within nested family, peer, school, community, cultural, and linguistic contexts (García Coll et al., 1996; Knight et al., 2017), and caregiver responses to an LGBTQ+ child's identity are embedded within these contexts. Consistent with IPARTheory (Rohner, 2021), caregiver acceptance and rejection represent core relational processes that shape youth development and may be expressed differently across cultural and linguistic contexts. Thus, validating the LCAS in Spanish is not only a matter of accessibility; it also allows researchers to examine how caregiver acceptance is expressed within Spanish‐speaking Latinx families.

Developmental ecological frameworks posit that identity formation among youth, including those who are LGBTQ+, unfolds within nested contexts of family, peer, school, community, culture, and language (García Coll et al., 1996; Knight et al., 2017). Consistent with contemporary developmental identity scholarship, youths' identities are shaped through ongoing transactions within culturally specific contexts and meaning systems (Galliher et al., 2017), particularly during adolescence when identity development and disclosure processes are most salient (Russell & Fish, 2019). For Latinx youth in particular, family processes are deeply imbued with cultural values (e.g., *familismo, respeto*), language socialization, and migration experiences (Abreu et al., 2020; Calzada et al., 2010). Spanish may serve as both a communication medium and the language in which core family and cultural values are learned and enacted (see review in Vázquez et al., 2024). English‐administered items may lose nuance or fail to capture meaning in Spanish‐speaking households, particularly around concepts such as honor or family image (see the review in Young et al., 2024). For these reasons, the present study did not assume that the English‐language LCAS factor structure would replicate exactly in Spanish. Instead, we examined whether the translated items formed a coherent, reliable, and culturally meaningful structure among Spanish‐speaking Latinx caregivers.

### Caregiver acceptance in English‐ and Spanish‐speaking Latinx households

Research comparing English‐ and Spanish‐speaking Latinx families shows meaningful differences in parenting attitudes, behaviors, disclosure practices, and cultural strain (Ramos et al., 2023). Within LGBTQ+ family contexts, these differences may shape how caregivers respond to a child's sexual orientation, gender identity, and gender expression. Qualitative research suggests that Spanish‐speaking caregivers may express strong emotional support while simultaneously navigating concerns about stigma, family reputation, and community perceptions, sometimes resulting in protective forms of concealment (Abreu et al., 2020; Abreu, Barrita, et al., 2025; Abreu, Lefevor, et al., 2023; Barrita, Parmenter, et al., 2025; Barrita, Abreu, et al., 2025; Barrita, Abreu, Carbajal, et al., 2026; Barrita, Abreu, Zelaya, et al., 2026). Accordingly, English‐language measures may not fully capture how bilingual and Spanish‐speaking families negotiate support, protection, and disclosure. Differences in language use may also influence how caregivers interpret survey items, potentially introducing measurement inequivalence and altering the meaning of key constructs related to acceptance and restriction (Van de Vijver & Leung, 1997). Therefore, validating the LCAS in Spanish is necessary to determine whether the measure captures both shared and language‐specific dimensions of LGBTQ+‐related caregiver acceptance among Latinx families.

### Culturally grounded approaches to measuring Latinx parenting

Family and developmental sciences emphasize that instruments must be culturally grounded, not merely linguistically translated, and represent constructs meaningful to specific cultural groups (Parra‐Cardona et al., 2012). Such approaches move away from deficit‐oriented assumptions and instead emphasize strengths within Latinx families, including expressions of love, protection, and belonging (Cahill et al., 2021). Latinx parenting is shaped by culturally embedded value systems, including familismo, respeto, simpatía, and personalismo, that influence parenting goals and family relationships (Cahill et al., 2021; Halgunseth et al., 2006). Familismo emphasizes family interconnectedness, loyalty, and mutual obligation; respeto emphasizes expectations around proper conduct and family dignity; and simpatía and personalismo emphasize warmth and relational harmony (Calzada et al., 2010, 2012; Halgunseth et al., 2006). These values vary across families based on national origin, generational status, and language use (Abreu, Lefevor, et al., 2023; García Coll et al., 1996). Within Latinx LGBTQ+ family contexts, these values may shape how caregivers balance acceptance, protection, and family cohesion, underscoring the importance of interpreting caregiver behaviors within their broader cultural context (Abreu, Gattamorta, et al., 2023; Abreu, Lefevor, et al., 2023). Accordingly, culturally grounded measurement is essential for capturing both shared and culturally specific expressions of caregiver acceptance and restriction among Latinx families. Such an approach supports the identification of asset‐based caregiving practices while also assessing behaviors that may place LGBTQ+ youth at risk, providing a better understanding of caregiver acceptance within Latinx cultural contexts.

### Current study: Why a Spanish adaptation of the LCAS is needed

Although prior literature highlights the importance of culturally and linguistically grounded measurement, no validated Spanish version of the LCAS currently exists for Spanish‐speaking Latinx caregivers of LGBTQ+ youth. Building on this work, the present study aimed to translate and validate a Spanish‐language version of the LCAS (LCAS‐S) using a Latinx caregiver sample. Given the potential for cultural and linguistic differences in how caregiver acceptance is expressed, we did not assume that the original English LCAS's factor structure would replicate exactly in Spanish. Accordingly, we employed an exploratory factor analysis (EFA) to examine the underlying structure of the translated items, followed by a confirmatory factor analysis (CFA) in an independent subsample to evaluate model fit.

The study had two primary aims: (Aim 1) we examined the factor structure and internal consistency reliability of the LCAS‐S; (Aim 2) we evaluated its convergent validity with measures of general caregiver acceptance, family cohesion and conflict, and homonegativity. We hypothesized that the LCAS‐S would demonstrate strong internal consistency and a multidimensional factor structure reflecting both affirming and restrictive caregiver responses. We also expected affirming LCAS‐S dimensions to be positively associated with family cohesion and general caregiver acceptance and negatively associated with family conflict and homonegativity. Finally, we expected restrictive LCAS‐S dimensions to be positively associated with family conflict and homonegativity and negatively associated with family cohesion and general caregiver acceptance.

## METHOD

### Participants

To qualify for the study, participants had to be Latinx parents or caregivers of LGBTQ+ youth living in the United States (either currently or someone who cared for an LGBTQ+ person during their adolescence), speak Spanish, and be aware of their child's sexual orientation and gender identity or gender expression. Because the LCAS was designed to assess caregiver acceptance of LGBTQ+ people during adolescence, participants were eligible if they currently cared for an LGBTQ+ youth or were reporting retrospectively on caregiving during the LGBTQ+ person's adolescence. Given the lack of agreement among behavioral science scholars regarding what constitutes “youth,” including distinctions based on biological stage, psychological processes, sociocultural transitions, and historical context (Sawyer et al., 2018), we did not impose a strict age range for the LGBTQ+ person's current age.

The study participants were 256 Spanish‐speaking Latinx caregivers (*M*_age_ = 37.32; *SD*_age_ = 8.00). Caregiver demographics are reported in Table 1. Across the sample, over half (70.7%) were women, 27.7% were men, and 1.2% were non‐binary. More than half (54%) were foreign‐born, and 46% were US‐born. Across ethnoracial identity, 70% self‐identified as White‐Latinx, 10% as Black‐Latinx, 14% as Indigenous‐Latinx, and 3% as Asian‐Latinx. Participants represented diverse Latinx national origins and were born in 15 countries. The largest national‐origin groups included individuals born in the United States (46%), Mexico (20%), Puerto Rico (12%), and Cuba (6%), with all other countries each representing less than 5% of the sample. The characteristics of their LGBTQ+ youth, as reported by caregivers, are included in Supplemental Table 1. Only caregivers completed the survey; LGBTQ+ youth were not surveyed, and the study was not dyadic.

**TABLE 1:** Caregiver demographics (*N* = 256).

Demographic variable	*N*	%
Age (*M*, *SD*)	37.32	8
Gender identity		
Woman	181	70.7
Man	71	27.7
Non‐binary	3	1.2
Nativity		
Born in US	117	45.7
Foreign Born	139	54.3
Race		
White‐Latino	178	69.5
Black‐Latino	26	10.2
Asian‐Latino	7	2.7
Indigenous‐Latino	35	13.7
Other	23	9.0
Religion		
Roman‐Catholic	94	36.7
Other Christian	93	36.3
Other	9	3.5
None	55	21.5
Employment status		
Employed	188	73.4
Non‐employed	68	26.6

### Procedures

Data were collected through Qualtrics Research Panels. The Qualtrics online survey included an IRB‐approved electronic consent form (Abreu, Gattamorta, et al., 2023) and survey instruments. Qualtrics Research Panels used specialized targeting techniques to distribute the survey to a prearranged pool of respondents who had agreed to be contacted by a marketing research service for studies for which they met recruitment criteria based on factors such as demographic information, life experiences, and unique skills. Qualtrics anonymously distributed the survey link to eligible participants, tracked survey completion for compensation purposes, and screened responses for eligibility and data quality, including procedures to identify fraudulent, duplicate, or bot responses. As a result, the final dataset provided by Qualtrics comprised eligible caregivers who met the study inclusion criteria, and no additional participants were removed by the research team after data cleaning. The survey was launched on June 11, 2021, and closed on February 9, 2023. Participants took an average of 44 min to complete the survey. The extended data‐collection period reflected the challenges of recruiting a hard‐to‐reach population with narrow inclusion criteria, as well as temporary pauses in recruitment due to limited financial resources for participant compensation. Participants received compensation through the Qualtrics panel system in accordance with panel‐provider policies.

### Instruments

#### Demographics

For this study, the Center for Latino Health and Research Opportunities demographic form (CLARO; El Centro‐University of Miami, n.d.) was used. This form includes 27 items regarding participants' characteristics and those of their LGBTQ+ children. Demographics included race, ethnicity, national origin, age, country of residence, income, education level, religion, employment status, relationship status, sexual orientation, and gender identity.

#### Latinx LGBTQ+ caregiver acceptance

Participants completed the Spanish version of the LCAS (Abreu, Gattamorta, et al., 2023). The LCAS consisted of 40 items that assessed the caregiver's acceptance and rejection of their LGBTQ+ child in Latinx families. Participants rated items on a 5‐point Likert Scale, ranging from total agreement (1) to total disagreement (5), with higher scores indicating greater acceptance. Because several items are reverse‐scored, items were coded so that higher scores reflected greater acceptance before computing total and subscale scores. Spanish‐language items are provided in Table 2, alongside English item content and retained LCAS‐S factor placement.

**TABLE 2:** Factor analyses.

Factor	Item Spanish	Item English	EFA extraction	CFA Std loadings
1‐ Concealment and Respeto [Ocultamiento y Respeto] (All reverse‐coded)	Deseo que mi hijo mantenga su identidad LGBTQ en secreto para proteger a la familia	I wish my child would keep their LGBTQ identity a secret to protect the family	0.785	0.831
	Mi hijo debe mantener su identidad LGBTQ en secreto para ser considerado parte de la familia	My child must keep their LGBTQ identity a secret in order to be considered part of the family	0.789	0.799
	Proteger la imagen de la familia es más importante que permitir que mi hijo exprese su orientación sexual o identidad de género	Protecting the image of the family is more important than allowing my child to express their sexual orientation or gender identity	0.794	0.732
	Tener en cuenta las opiniones de la familia es más importante que ser abiertamente gay, LGBTQ	Taking into account the opinions of the family is more important than being openly LGBTQ	0.761	0.715
	Preferiría no revelar su orientación sexual o identidad de género a los miembros de mi comunidad (por ejemplo, sacerdotes, pastores, miembros de mi iglesia, vecinos) que no lo apoyarán	I would prefer not to reveal my child's sexual orientation or gender identity to members of my community (for example, priests, pastors, members of my church, neighbors) that would not support them	0.679	0.705
	Ser abiertamente LGBTQ es irrespetuoso con la familia	Being openly LGBTQ is disrespectful to the family	0.663	0.820
	Actuar “gay” es irrespetuoso	Acting “gay” is disrespectful	0.584	0.661
	Siento que tengo que decidir entre mi hijo LGBTQ y el resto de mi familia	I feel that I have to choose between my LGBTQ child and the rest of my family	0.753	0.808
	Desearía que mi hijo no revelara su orientación sexual o identidad de género a amigos de la familia	I wish my child would not reveal their sexual orientation or gender identity to family friends	0.571	0.883
2‐ Supportive Actions [Acciones de Apoyo]	He invitado a la(s) pareja(s) de mi hijo a participar en actividades familiares	I have invited my child's romantic partner(s) to participate in family activities	0.798	0.865
	He invitado a la(s) pareja(s) de mi hijo a participar en asuntos familiares	I have invited my child's romantic partner(s) to participate in family matters	0.634	0.835
	Elegí a mi hijo(a/e) LGBTQ sobre mi familia	I chose my LGBTQ child over my family	0.664	0.529
	Participaría en eventos de Orgullo LGBTQ para mostrar apoyo a mi hijo LGBTQ	I would participate in LGBTQ Pride events to show support to my LGBTQ child	0.634	0.589
3. Public Opinion [Opinión Pública] (All reverse‐coded)	He consultado con mi sacerdote/pastor/líder espiritual sobre cómo tratar con mi hijo LGBTQ	I have asked my priest/pastor/spiritual leader about how to deal with my LGBTQ child	0.808	0.558
	Le pedí consejo a otras personas importantes para mi sobre cómo tratar con mi hijo LGBTQ	I have asked other people whose opinion is important to me for advice about how to deal with my LGBTQ child	0.571	0.616
	Le pedí a mi hijo LGBTQ que no actúe “gay” frente a miembros de mi comunidad (por ejemplo, sacerdote, pastor, miembros de mi iglesia, vecinos)	I have asked my LGBTQ child not to act “gay” in front of members of my community (for example, priests, pastors, members of my church, neighbors)	0.575	0.878
4. Fear of Family Rejection [Miedo al Rechazo Familiar] (All reverse‐coded)	Me preocupa lo que dirá mi familia si descubren que mi hijo es LGBTQ	I am worried about what my family will say if they find out that my child is LGBTQ	0.708	0.874
	Me preocupa que mi familia rechace a mi hijo LGBTQ	I am worried that my family would reject my LGBTQ child	0610	0.703
	Me preocupa que mi familia me rechace porque tengo un hijo LGBTQ	I am worried that my family would reject me because I have an LGBTQ child	0.751	0.813
	Me preocupa que la orientación sexual de mi hijo cree conflictos en la familia	I am worried that my child's sexual orientation could cause conflicts in the family	0.753	0.817
	Me preocupa que mi familia me pida decidir entre mi hijo LGBTQ y el resto de mi familia	I am worried that my family would ask me to choose between my LGBTQ child and the rest of my family	0.828	0.803
5. Affirming LGBTQ child's place in family [Afirmación del Lugar del Hijo/a/e LGBTQ+ en la Familia]	Está bien que mi hijo LGBTQ asista a actividades familiares	It is fine for my LGBTQ child to attend family activities	0.676	0.737
	Aceptar a mi hijo LGBTQ es una manera de mantener a la familia unida	Accepting my LGBTQ child is a way of keeping the family united	0.695	0.678
	Consideraría a los hijos de mi hijo LGBTQ como mis nietos	I would consider my LGBTQ child's children to be my grandkids	0.715	0.605
6. Outness [Visibilidad LGBTQ+]	Está bien que mi hijo LGBTQ haya revelado su identidad a otros miembros de la familia	It was fine that my LGBTQ child revealed their identity to other family members	0.698	0.789
	Está bien que mi hijo LGBTQ haya revelado su identidad a amigos de la familia	It was fine that my LGBTQ child revealed their identity to family friends	0.833	0.932
	Está bien que mi hijo LGBTQ haya revelado su identidad a los miembros de mi comunidad (por ejemplo, sacerdote, pastor, miembros de mi iglesia, vecinos)	It was fine that my LGBTQ child revealed their identity to the members of my community (for example, priests, pastors, members of my church, neighbors)	0.667	0.785

*Note*: *N* = 100 (EFA); 156 (CFA).

### Translation and backtranslation

The LCAS was translated using established guidelines for adapting research instruments across cultures (Brislin, 1970). First, one of the authors, a bilingual psychologist with translation experience, translated the measure from English into Spanish. Then another bilingual author independently back‐translated it into English. All authors reviewed and discussed any discrepancies between the original and back‐translated versions until reaching a consensus. This process resulted in an initial 40‐item version of the LCAS‐Spanish (LCAS‐S), which was tested and validated in this study.

The research team developed the test items using a blend of Mexican American and Caribbean American Spanish (e.g., Cuban, Puerto Rican), reflecting the predominant national‐origin backgrounds of the sample and dialects commonly used within Latinx communities in the study region, while avoiding highly regional phrases that might not be easily understood by Spanish‐speaking caregivers from other Latin American backgrounds. Because of bias, or a systematic influence that can distort respondents' scores (Peña, 2007), we paid particular attention to avoiding idiomatic expressions or regionalisms that might confuse Latinx participants. First‐generation Latinx immigrant authors completed the initial translation, while bilingual team members of Latinx heritage conducted the back‐translation and subsequent revisions. Several authors have expertise in psychometrics and Spanish‐language instrument adaptation. The team prioritized conceptual equivalence over literal translation, especially for LGBTQ+ identity terms and family‐context items. For example, the term “gay” was retained in Spanish because it is widely used and recognizable across Spanish‐speaking LGBTQ+ communities, whereas other terms were reviewed to ensure they were understandable and not overly regional. Because translation decisions were resolved by consensus among bilingual experts rather than independent item ratings, interrater reliability statistics such as Cohen's kappa were not calculated.

#### General caregiver acceptance

General caregiver acceptance was assessed using the Spanish version of PARQ–Short Form (Rohner & Ali, 2020), which captures the extent to which caregivers are perceived as warm, affectionate, and supportive. Items (e.g., “My caregiver makes me feel loved”) are rated on a 4‐point Likert‐type scale ranging from (0) *almost always true* to (4) *almost never true* and summed to create a total score. Higher scores reflect greater perceived caregiver acceptance, including consistent warmth, responsiveness, and emotional support. In this study, the measure demonstrated strong reliability (*α* = 0.85).

#### Family cohesion/conflict

Family cohesion and conflict were measured using the Spanish version of the 18‐item Family Environment Scale (FES; Moos & Moos, 1994), which assesses perceptions of family functioning across two 9‐item subscales: Cohesion and Conflict. Participants rated each statement as *true* (1) or *false* (0). The Cohesion subscale captures supportive, connected, and cooperative family interactions (e.g., “Family members really help and support one another”). The Conflict subscale captures openly expressed anger, disagreement, and conflict within the family. The average item score for each subfactor, with higher cohesion scores indicating greater emotional bonding and higher conflict scores indicating greater family conflict, was used to assess validity. In this study, the measure demonstrated strong reliability for both cohesion (*α* = 0.88) and conflict (*α* = 0.86).

#### Homonegativity

Homonegativity was assessed using a modified Spanish version of the Modern Homonegativity Scale (MHS; Morrison & Morrison, 2002). The LCAS development study (Abreu, Gattamorta, et al., 2023) adapted the MHS measure to capture negative attitudes toward LGBTQ+ people broadly, resulting in a 12‐item scale. Participants rated each statement on a 5‐point Likert scale ranging from *strongly disagree* (1) to *strongly agree* (5; e.g., “LGBTQ+ people often exaggerate the challenges they face.”). Items were summed, with higher scores indicating stronger negative attitudes toward LGBTQ+ individuals. In this study, the MHS demonstrated strong reliability (*α* = 0.91).

### Data analysis

First, we conducted a thorough data‐cleaning process to ensure data quality. Qualtrics employs multiple safeguards to detect and remove low‐quality or invalid responses, including bots, duplicate entries, speeders, and fraudulent submissions. The platform uses Google's invisible reCAPTCHA, response‐quality screening, data encryption, and completion rates, among other measures, to identify and flag suspicious activity. Qualtrics Research Panels screened respondents for eligibility and data quality before providing the final dataset to the research team. Data analysis for this study was conducted using Statistical Package for the Social Sciences and RStudio with the *“lavaan”* package (Rosseel, 2012). Before conducting factor analyses, we examined the data for missing values, normality, and outliers. Items were inspected for skewness, kurtosis, and distributional properties to ensure suitability for factor analysis. Item‐level descriptive statistics, including means, standard deviations, and score ranges, were examined to assess variability and potential range restriction. Sampling adequacy was evaluated using the Kaiser–Meyer–Olkin (KMO) measure and Bartlett's test of sphericity (Tabachnick & Fidell, 2001).

We followed a validation process similar to that used in the LCAS (see Abreu, Gattamorta, et al., 2023). Consistent with recommended procedures for cross‐cultural adaptation of psychological measures (Beaton et al., 2000; Sousa & Rojjanasrirat, 2011), we used a two‐step analytic approach to evaluate the LCAS‐S. Because translation and cultural context can influence how items are interpreted and how they relate to one another (Peña, 2007), we did not assume that the English factor structure would replicate exactly in Spanish. This decision was also consistent with the study aims, which were to examine whether Spanish‐language administration produced both shared and culturally or linguistically distinct dimensions of LGBTQ+‐related caregiver acceptance. Accordingly, using all 40 translated LCAS items, we conducted an EFA prior to a CFA to identify the underlying factor structure of the LCAS‐S and evaluate whether the original English‐language factor structure replicated in the Spanish‐language version.

To evaluate the factor structure, the total sample (*N* = 256) was divided into two subsamples: 100 for the EFA and 156 for the CFA. This allowed independent testing of exploratory and confirmatory models, reducing the risk of capitalizing on chance. Subsample sizes followed recommended guidelines for factor analysis, suggesting at least 100 participants for stable factor recovery (Worthington & Whittaker, 2006). EFA was conducted using principal axis factoring with direct oblimin rotation to allow for correlated factors, aligning with psychometric recommendations for assessing whether a theoretical structure holds in a new linguistic or cultural group (Fabrigar et al., 1999; Worthington & Whittaker, 2006). This approach was consistent with the original LCAS validation study, which also used exploratory factor analytic procedures to identify the latent structure of caregiver acceptance items; however, the present study extended this approach by evaluating the translated item set in Spanish and then testing the resulting structure through CFA in an independent subsample. To determine factor retention, eigenvalues from the observed data were compared with those from a randomly generated dataset of equal size and dimensionality (Hayton et al., 2004). We also examined the scree plot and parallel analysis results to guide factor retention. Factors were retained if eigenvalues exceeded the random data, included at least three items, and demonstrated conceptual interpretability (Worthington & Whittaker, 2006). Factor extraction followed sequential criteria: (a) primary loadings ≥0.40, (b) no cross‐loadings >0.30, and (c) a difference ≥0.15 between primary and secondary loadings (Worthington & Whittaker, 2006). When statistical criteria and conceptual interpretation diverged, the research team reviewed the item content to determine whether the factor represented a coherent and theoretically meaningful dimension of LGBTQ+‐related caregiver acceptance.

Based on the results of the EFA, we then performed a CFA to test the fit of the identified model, evaluating model adequacy with multiple indices (e.g., CFI, TLI, RMSEA, SRMR) in accordance with factor analysis recommendations and guidelines for cross‐cultural validation (Fabrigar et al., 1999; Worthington & Whittaker, 2006). We interpreted model fit using commonly applied benchmarks: CFI and TLI values of .90 or higher, RMSEA and SRMMR values of .08 or lower, as indicating acceptable fit. Modification indices were reviewed for diagnostic purposes; however, no post hoc correlated errors or additional model modifications were retained to avoid overfitting the model to this sample. To further validate the LCAS‐S, we established convergent validity by computing bivariate correlations between LCAS‐S subscales and total scores, and between LCAS‐S total scores and PARQ total, FES‐Cohesion, FES‐Conflict, and homonegativity.

## RESULTS

Before conducting the EFA, we assessed the data's suitability for factor analysis. Bartlett's test of sphericity was significant, *χ* ^2^(406) = 1928.43, *p* < .001, and the KMO measure of sampling adequacy was 0.89, both indicating that the correlation matrix was factorable and appropriate for EFA (Tabachnick & Fidell, 2001). Item responses demonstrated adequate variability across the full range of response options (1–5). Item means ranged from 1.49 to 4.19 (*M* = 3.19, *SD* = 0.88), suggesting no substantial restriction of range.

### EFA

Results from the eigenvalues, scree plot, and parallel analysis supported a seven‐factor solution for all 40 items, which accounted for 71.89% of the total variance: 39.35% from the first factor, 12.80% from the second, 3.76% from the third, 3.48% from the fourth, 2.67% from the fifth, 2.16% from the sixth, and 1.84% from the seventh. However, we evaluated the factor–item structure based on our established item retention criteria (Worthington & Whittaker, 2006), which resulted in four items removed due to failing to meet the minimum loading threshold of .40, and four items removed due to exhibiting cross‐loadings exceeding .30 or differences between their primary and secondary loadings smaller than .15. A seventh factor and its five items were also removed because, despite demonstrating internal consistency, the factor reflected heterogeneous themes related to restriction, family discomfort, and anticipated social judgment that lacked sufficient conceptual coherence and theoretical distinctiveness to represent an interpretable dimension of LGBTQ+‐related caregiver acceptance among Spanish‐speaking Latinx caregivers (see Supplemental Table 2 for a full list of items removed). After these steps, the final solution comprised six factors and 27 items, reflecting a coherent and theoretically interpretable structure. Table 2 presents the pattern matrix with the retained items and corresponding factor loadings.

The final EFA solution yielded six factors for the LCAS‐S that both overlapped with and diverged meaningfully from the original English‐language LCAS structure (Abreu, Gattamorta, et al., 2023). Specifically, two factors retained themes similar to the original LCAS, although with fewer items retained: Supportive Actions (LCAS‐S = four items; LCAS = five items) and Outness (LCAS‐S = three items; LCAS = ten items). Four new factors emerged for the LCAS‐S: Concealment and Respeto (nine items), Public Opinion (three items), Fear of Family Rejection (five items), and Affirming the LGBTQ+ Child's Place in the Family (three items). Supplemental Table 2 provides a detailed comparison between items for LCAS‐S versus those in the LCAS.

Factor 1, Concealment and Respeto (*α* = .94; *R* ^2^ = .394) included nine items with factor loadings ranging from .571 to .794. This factor reflects caregivers' beliefs about secrecy, family image, and respectability in relation to their child's LGBTQ+ identity. Items emphasized concealment within the family and broader community, prioritizing family reputation and perceiving LGBTQ+ behavior openly as disrespectful.

Factor 2, Supportive Actions (*α* = .82; *R* ^2^ = .128), consisted of four items with factor loadings ranging from .634 to .798. Items captured caregivers' active efforts to support their LGBTQ+ child, including inviting the child's romantic partners to family events, involving them in family matters, participating in LGBTQ Pride activities, and prioritizing their child over extended family expectations. This factor reflects concrete, observable behaviors that communicate family support.

Factor 3, Public Opinion (*α* = .81; *R* ^2^ = .038), included three items with factor loadings ranging from .571 to .808. Items reflected caregivers' concerns about and responsiveness to external social influences, such as seeking guidance from religious or respected community figures and managing how their child behaves in the community. This factor captures the role of external social surveillance and community norms in shaping family responses to a child's LGBTQ+ identity.

Factor 4, Fear of Family Rejection (*α* = .90; *R* ^2^ = .035), was composed of five items with factor loadings ranging from .610 to .828. Items described caregivers' anxieties about how their family might respond to their child's LGBTQ+ identity, including fears of rejection directed at the child, at themselves, or at the broader family unit. This factor reflects anticipated conflict and rejection within the family system stemming from the child's identity.

Factor 5, Affirming the LGBTQ Child's Place in the Family (*α* = .82; *R* ^2^ = .027), consisted of three items with factor loadings ranging from .676 to .715. Items highlighted attitudes affirming the child's belonging within the family, such as supporting their participation in family activities, viewing acceptance as essential to family unity, and embracing future grandchildren regardless of the child's sexual orientation or gender identity. This factor reflects a family‐inclusive, affirming orientation.

Factor 6, Outness (*α* = .87; *R* ^2^ = .022), included three items with factor loadings ranging from .667 to .833. Items focused on caregivers' comfort with their child openly disclosing their LGBTQ+ identity to family, friends, and community members. This factor reflects endorsement of visibility and authenticity across interpersonal and community contexts. Together, the LCAS‐S, as a six‐factor measure (*α* = .89; *R* ^2^ = .701), represents a multidimensional structure that captures both restrictive and affirming dimensions of Spanish‐speaking caregivers' responses to their LGBTQ+ child.

### CFA

A CFA was conducted to evaluate the fit of the six‐factor structure identified in the exploratory phase. The hypothesized model demonstrated an acceptable overall fit to the data, *χ* ^2^(302) = 584.15, *p* < .001, CFI = .906, TLI = .901, RMSEA = .077 (90% CI [.068, .087]), and SRMR = .068. Collectively, these indices met or approached the recommended cutoffs (Hooper et al., 2008) for acceptable model fit (CFI, TLI ≥ .90), supporting the adequacy of the six‐factor solution for the LCAS‐S. Standardized factor loadings for the final CFA model are presented in Table 2 and ranged from moderate to strong across factors, supporting the adequacy of the retained items for representing their intended latent constructs. No additional items were removed following the CFA, and no post hoc model modifications (e.g., correlated residuals) were added. Overall, the CFA findings supported the adequacy and stability of the six‐factor LCAS‐S model.

### Validity analyses

Descriptive statistics for all LCAS‐S subscales and validation measures are presented in Table 3. Validation measures demonstrated adequate variability across constructs. Consistent with our hypotheses, bivariate correlations supported the convergent validity of the LCAS‐S subscales and total score. Restrictive dimensions (e.g., Concealment and Respeto, Public Opinion, and Fear of Family Rejection) were positively intercorrelated, whereas affirming dimensions (e.g., Supportive Actions, Affirming the LGBTQ+ Child's Place in the Family, and Outness) were positively associated with one another and negatively associated with restrictive dimensions. Correlations with external validation measures further supported construct validity. Higher LCAS‐S total scores were associated with greater family cohesion and lower family conflict. Restrictive LCAS‐S dimensions were associated with greater family conflict, lower family cohesion, and higher homonegativity, whereas affirming dimensions were associated with the opposite pattern. Associations with the PARQ were generally smaller but occurred in theoretically expected directions. Collectively, these findings provide evidence of convergent validity for the LCAS‐S and support the distinction between affirming and restrictive dimensions of LGBTQ+‐related caregiver acceptance among Spanish‐speaking Latinx caregivers.

**TABLE 3:** Correlations between the LCAS‐S Subscales and other measures for validity.

Measures	Concealment & respect	Supportive actions	Public opinion	Fear of rejection	Affirming LGBTQ child's place	Outness	LCAS‐S total
Concealment & respect	–	−.286**	.593**	.747**	−.433**	−.391**	.797**
Supportive actions	−.286**	–	−.163**	−.231**	.438**	.607**	.607**
Public opinion	.593**	−.163**	–	.564**	−.259**	−.199**	.657**
Fear of rejection	.747**	−.231**	−.564**	–	−.359**	−.333**	.629**
Affirming LGBTQ child	−.433**	.438**	−.259**	−.359**	–	.524**	.746**
Outness	−.391**	.607**	−.199**	−.333**	.524***	–	.620**
PARQ	−.137**	.142**	−.106*	−.145**	.093*	.113**	.108**
Family cohesion	−.384**	.199**	−.250**	−.326**	.366**	.217**	.293**
Family conflict	.333**	−.148*	.218**	.365**	−.234**	−134**	−.339**
Modern homonegativity	.208**	.125*	.148**	.070*	−.108**	−.102**	−.104*

*Note*: LCAS‐S, LGBTQ Caregiver Acceptance Scale‐Spanish; PARQ, Parental Acceptance‐Rejection Questionnaire.

* *p* < .05.

** *p* < .01.

*** *p* < .001.

## DISCUSSION

The present study reports on the translation and validation of the LCAS‐S, contributing to culturally grounded assessment tools for Spanish‐speaking Latinx families who have historically been underrepresented in measurement‐based research. Guided by cross‐cultural adaptation principles (Beaton et al., 2000; Peña, 2007) and the theoretical foundation of the LCAS (Abreu, Gattamorta, et al., 2023), our findings support a Spanish‐language version that captures the complex, culturally embedded patterns through which Spanish‐speaking caregivers respond to their LGBTQ+ children. Consistent with the study aims and hypotheses, the LCAS‐S demonstrated strong reliability, acceptable model fit, and theoretically expected associations with family functioning, general caregiver acceptance, and homonegativity. The findings also suggest that caregiver acceptance and rejection are shaped by culturally salient relational processes, including respeto, familismo, and concerns regarding extended family and community expectations within many Spanish‐speaking Latinx households. In line with extensive research emphasizing the protective role of caregiver acceptance for LGBTQ+ youth well‐being (Abreu, Barrita, et al., 2025; Ancín‐Nicolás et al., 2024; Barrita, Parmenter, et al., 2025; DelFerro et al., 2024; Ryan et al., 2010), the LCAS‐S offers a meaningful step toward ensuring that Spanish‐speaking families are equitably represented in empirical work and in clinical practice. Although the present study did not examine adolescent developmental outcomes directly, the LCAS‐S provides a culturally responsive tool for future research examining how caregiver responses during adolescence relate to LGBTQ+ youths' identity development, belonging, and psychological adjustment (Russell & Fish, 2019).

### LCAS‐S adaptation in relation to the LCAS

Consistent with the LCAS (Abreu, Gattamorta, et al., 2023), the LCAS‐S demonstrated strong reliability and a coherent factor structure. However, the thematic configuration of the factors differed in important ways from the English version (Supplemental Table 2). The six factors identified, Concealment and Respeto, Supportive Actions, Public Opinion, Fear of Family Rejection, Affirming the Child's Place in the Family, and Outness, collectively represent a blend of restrictive and affirming responses consistent with Latinx cultural frameworks (Cahill et al., 2021; García Coll et al., 1996; Halgunseth et al., 2006). The prominence of the Concealment and Respeto factor is especially noteworthy because secrecy, family image, and respect emerged as a unified relational dimension among Spanish‐speaking caregivers rather than as separate constructs, as observed in the English‐language LCAS. This pattern reflects research showing that respeto and family reputation play central roles in Latinx parenting (Calzada et al., 2010; Halgunseth et al., 2006), particularly when caregivers navigate tensions between cultural values and an LGBTQ+ child's visibility (Abreu, Lefevor, et al., 2023). From a developmental perspective, these tensions may become especially salient during adolescence, when LGBTQ+ youth are navigating identity disclosure while remaining deeply embedded within family and community systems (Russell & Fish, 2019). Thus, concealment may not always reflect simple rejection, but rather a culturally situated strategy to negotiate family dignity, safety, and social belonging within broader relational networks.

Several LCAS factors (Abreu, Gattamorta, et al., 2023), such as Supportive Actions and Outness, replicated in the LCAS‐S, indicating that some dimensions of caregiver acceptance are consistent across English‐ and Spanish‐speaking Latinx caregivers. However, the emergence of factors such as Public Opinion and Fear of Family Rejection highlights the unique sociocultural pressures faced by Spanish‐speaking caregivers, many of whom navigate extended family networks, religious communities, and immigrant or bicultural contexts where conformity and social surveillance may be especially salient (Ramos et al., 2023). These themes echo qualitative work demonstrating that Latinx caregivers may simultaneously express profound love for their LGBTQ+ children while balancing concerns about community judgment, family cohesion, and broader cultural expectations regarding public visibility and family reputation (Abreu et al., 2020). These findings also align with emerging research on collective and within‐culture minority stressors among LGBTQ+ people of color, including pressures related to family reputation, community surveillance, and *qué dirán* concerns (Estevez et al., 2025; Parmenter et al., 2026), which may shape how caregivers negotiate acceptance and disclosure. At the same time, the factor Affirming the Child's Place in the Family underscores that many Spanish‐speaking caregivers hold strong beliefs regarding unconditional belonging and family unity, consistent with familismo and relational cultural values emphasizing connection, support, and interdependence (Cahill et al., 2021; García Coll et al., 1996; Halgunseth et al., 2006). Together, these findings reinforce that caregiver responses to LGBTQ+ youth are multidimensional and often involve simultaneous experiences of support, protection, fear, and negotiation within culturally embedded family systems.

From the LCAS (Abreu, Gattamorta, et al., 2023), several items were removed. This outcome is expected in cross‐cultural adaptation, where items that function well in one language may not translate conceptually or psychometrically in another (Peña, 2007; Van de Vijver & Leung, 1997). Some items may have been less culturally salient or less conceptually cohesive for Spanish‐speaking caregivers. For example, questions about LGBTQ+ advocacy or imagining future queer parenting might seem more abstract or less connected to the lived experiences of caregivers in more traditional or community‐focused contexts. Other items may have lost subtle distinctions in translation into Spanish, particularly when English terms rely on idiomatic or culturally embedded phrasing. Nevertheless, the removal of items resulted in a coherent factor structure, suggesting that the LCAS‐S taps into culturally salient constructs rather than methodological noise. Consistent with cross‐cultural measurement frameworks, these findings underscore that translation alone does not guarantee conceptual equivalence across linguistic and cultural groups (Beaton et al., 2000; Peña, 2007).

### Implications

The findings from this study have important implications for developmental research, clinical practice, and culturally grounded measurement development involving LGBTQ+ youth and their families. The LCAS‐S provides scholars with a culturally and linguistically responsive tool to assess how Spanish‐speaking Latinx caregivers navigate acceptance and rejection of their LGBTQ+ children, a population that has been significantly underrepresented in empirical studies despite comprising a large proportion of Latinx communities in the United States (Pew Research Center, 2022). By offering a validated Spanish‐language version of the LCAS, researchers may more accurately capture culturally embedded beliefs, relational processes, and caregiving behaviors that may not emerge through English‐only assessments. While other shorter scales that measure family dynamics and acceptance exist (e.g., PARQ–Short Form; Family Environment Scale [FES]; Moos & Moos, 1994; Rohner & Ali, 2020), these do not account for culturally grounded Latinx values captured by our LCAS‐S (e.g., respeto, familismo, simpatía, and personalismo; Calzada et al., 2012; Cahill et al., 2021; Halgunseth et al., 2006). Given the multidimensional nature of Latinx cultural values and family processes, the LCAS‐S may offer a more nuanced understanding of LGBTQ+‐specific caregiver acceptance and restriction than broader family functioning measures alone.

Clinically, the LCAS‐S offers providers a nuanced lens on the affirming and restrictive dynamics reflected in its items, including influences of respeto, extended family expectations, and broader community expectations within Spanish‐speaking households. These insights can improve intervention planning, enhance family‐based counseling approaches, and help practitioners tailor strategies in ways that honor caregivers' cultural values while promoting acceptance and reducing rejection (Abreu, Martin, et al., 2023; Abreu, Gattamorta, et al., 2025; Parra‐Cardona et al., 2012). Importantly, the LCAS‐S may also help clinicians and researchers move beyond binary conceptualizations of “accepting” versus “rejecting” caregivers by recognizing that many families simultaneously experience support, fear, protection, ambivalence, and negotiation as they respond to a youth's LGBTQ+ identity. This multidimensional perspective may be particularly valuable for future developmental research and family‐based practice during adolescence, when LGBTQ+ youth often remain emotionally, socially, and economically connected to caregivers while navigating identity development and disclosure processes (Russell & Fish, 2019). More broadly, the LCAS‐S advances measurement equity by increasing opportunities for Spanish‐speaking caregivers to participate in LGBTQ+ family research and by supporting culturally and linguistically responsive approaches to assessment, intervention, and family‐based care.

### Limitations and future studies

Despite these contributions, several limitations should be considered when interpreting findings. Although the overall sample size was within acceptable ranges for scale validation, dividing the dataset into EFA and CFA subsamples placed analyses near the lower boundary of recommended sample sizes for stable factor recovery (Worthington & Whittaker, 2006). Smaller sample sizes increase the likelihood that weaker items will fail to meet retention thresholds or yield unstable loadings, which may partly explain why some items from the LCAS were removed in the Spanish version. In addition, the EFA‐to‐CFA split‐sample design, although methodologically sound, reduces power within each subsample and may limit the generalizability or stability of emerging factor structures. Relatedly, the use of strict psychometric retention criteria (e.g., minimum loading thresholds) may have contributed to the removal of items that conceptually align with Latinx parenting but did not perform well in this specific dataset. Another limitation concerns the challenge of disentangling linguistic effects from demographic, socioeconomic, or cultural differences between the English‐ and Spanish‐speaking samples. Although several lines of evidence suggest that language meaningfully shaped the factor structure, including the emergence of culturally coherent factors and the alignment of results with known cultural parenting patterns (Nair et al., 2009), differences in nativity, acculturation, and educational backgrounds across samples may also have contributed to response patterns. Because formal tests of measurement invariance were not conducted, it is not possible to determine whether differences between the LCAS and LCAS‐S reflect linguistic variation, broader cultural processes, or demographic differences between samples. Consequently, the present findings should not be interpreted as supporting direct comparisons between English‐ and Spanish‐speaking Latinx caregivers, as foundational measurement equivalence has not yet been established. Future research should formally examine configural, metric, and scalar invariance using comparable English‐ and Spanish‐speaking Latinx samples before drawing conclusions about group differences. Although stable factors such as Supportive Actions and Outness emerged across both language versions, suggesting some common underlying dimensions (Gianola et al., 2025; Romero et al., 2004), additional work is needed to determine which aspects of caregiver acceptance are shared across languages and which reflect culturally or linguistically specific processes.

The translation and adaptation process also introduces inherent methodological challenges, even when conducted in accordance with best‐practice guidelines (Beaton et al., 2000). Some items may have lost subtle semantic meaning despite accurate translation, particularly those involving nuanced expressions of LGBTQ+ advocacy, future‐focused parenting expectations, or culturally specific conceptualizations of family support. The fact that certain items formed distinct factors in English but collapsed or shifted thematically in Spanish highlights the need to consider not only semantic equivalence but also cultural meaning and lived experience when validating instruments across languages (Peña, 2007). Additionally, online survey formats may reduce opportunities to clarify item meaning for respondents, which can disproportionately affect translated measures administered to linguistically diverse populations.

Several additional methodological limitations warrant consideration. First, only caregivers participated in the present study; LGBTQ+ youth were not surveyed, and the study was not dyadic. As a result, the findings reflect caregiver perspectives only and cannot capture how youth themselves experience or interpret caregiver acceptance and rejection. Second, because participants were caregivers who were aware of their child's LGBTQ+ identity and willing to participate in a study focused on LGBTQ+ caregiving, the sample may underrepresent caregivers with highly rejecting attitudes or limited awareness of their child's identity. At the same time, this recruitment context likely reflects an important feature of LGBTQ+ family research itself, as caregiver awareness and willingness to participate may represent meaningful dimensions of the caregiving experience rather than simple sampling bias. Another limitation is that the present study did not examine whether caregiver acceptance and rejection processes differed across caregivers of cisgender, transgender, and nonbinary LGBTQ+ youth. Given evidence that gender‐diverse youth may experience distinct family and cultural stressors within Latinx communities, the LCAS‐S may function differently across these groups in ways that were not assessed in the current study. Notably, the sample nevertheless demonstrated substantial variability across LCAS‐S items, suggesting that a broad range of affirming and restrictive caregiver responses was captured despite potential self‐selection effects. Finally, the cross‐sectional design limits conclusions regarding developmental or causal processes.

Future studies should recruit larger and more diverse samples of Spanish‐speaking Latinx caregivers of LGBTQ+ youth, including broader representation across national origins, racialized identities, immigration experiences, linguistic backgrounds, and geographic regions of the United States Larger samples would support more robust factor analytic procedures, permit cross‐validation of the six‐factor structure, and allow for formal tests of configural, metric, and scalar invariance between the English‐ and Spanish‐language versions of the LCAS. Future research would also benefit from qualitative and mixed‐methods approaches, including cognitive interviews or focus groups, to further examine how Spanish‐speaking caregivers interpret LCAS‐S items and whether the identified factors reflect caregivers' lived experiences across diverse Latinx communities. Such work could inform future item refinement and identify culturally meaningful expressions of support or restriction not fully captured by the current scale. Future research should examine whether LCAS‐S dimensions function similarly across caregivers of cisgender, transgender, and nonbinary LGBTQ+ youth, particularly given evidence that gender‐diverse youth may encounter distinct family, cultural, and minority stress processes within Latinx communities (Abreu, Sostre, et al., 2025). Future studies should also incorporate youth reports, dyadic family designs, and longitudinal methodologies to better understand how caregiver acceptance and rejection processes are experienced across family members and how these dynamics evolve over time. Longitudinal research may be especially important for examining whether LCAS‐S dimensions prospectively predict youth well‐being, family functioning, and caregiver adjustment across adolescence and emerging adulthood.

## CONCLUSION

In sum, the present study provides preliminary evidence that the LCAS‐S is a psychometrically sound and culturally grounded instrument that captures the multidimensional experiences of acceptance and rejection among Spanish‐speaking Latinx caregivers of LGBTQ+ youth. Although differences emerged between the English and Spanish versions, the findings highlight the importance of considering cultural and linguistic context when assessing caregiver acceptance among Latinx families. Future research should determine the extent to which these differences reflect language, culture, or broader measurement processes. By extending the LCAS (Abreu, Gattamorta, et al., 2023) into Spanish, this study contributes to a broader movement toward culturally and linguistically inclusive research tools that better reflect the diversity of Latinx families. Such tools are essential for advancing culturally responsive research, improving family‐based clinical practice, and supporting future research on the relational environments in which Latinx LGBTQ+ youth navigate identity development, belonging, and well‐being across adolescence and emerging adulthood.

## AUTHOR CONTRIBUTIONS

**Cristalís Capielo Rosario:** Conceptualization; writing – original draft; methodology. **Roberto L. Abreu:** Conceptualization; writing – original draft; writing – review and editing; methodology; investigation; formal analysis; data curation; supervision. **Aldo M. Barrita:** Conceptualization; investigation; writing – original draft; writing – review and editing; methodology; formal analysis; data curation; supervision. **Melanie Domenech Rodriguez:** Conceptualization; writing – original draft; methodology. **Kirsten A. Gonzalez:** Methodology; conceptualization; writing – original draft. **Alexa Parra:** Conceptualization; writing – original draft; methodology; data curation. **Karina A. Gattamorta:** Conceptualization; writing – original draft; investigation; methodology; data curation; supervision; formal analysis.

## FUNDING INFORMATION

The author(s) received no financial support for the research, authorship, and/or publication of this article.

## CONFLICT OF INTEREST STATEMENT

We have no known conflicts of interest to disclose.

## ETHICS STATEMENT

This study was conducted in accordance with research ethical principles and standards and was approved by the Institutional Review Board of the University of Miami on 1/7/22 (IRB #20200393).

## CONSENT STATEMENT

Participants were presented with an informed consent online form before participation.

## Supporting information


**Supplemental Table 1.** LGBTQ+ person demographics (reported by caregiver; *N* = 256).
**Supplemental Table 2**. LCAS versus LCAS‐S item comparison for placement or removal.

## Data Availability

The data that support the findings of this study are available from the authors upon reasonable request.

## References

[jora70254-bib-0001] Abreu, R. L. , Barrita, A. , Sostre, J. P. , Parmenter, J. , & Watson, R. (2025). Interest in *PrEP*, cyberbullying, internalized LGBTQ stigma, and parental support among Latinx sexual and gender diverse youth. Health Psychology, 44(3), 266–274. 10.1037/hea0001455 39992772

[jora70254-bib-0002] Abreu, R. L. , Gattamorta, K. A. , Gonzalez, K. A. , Capielo Rosario, C. , Roman Laporte, R. L. , & Domenech Rodríguez, M. M. (2023). LGBTQ Caregiver Acceptance Scale (LCAS): Development and validation with a Latinx sample. Journal of Family Psychology, 37(6), 875–887. 10.1037/fam0001122 37358526

[jora70254-bib-0003] Abreu, R. L. , Gattamorta, K. A. , Roman Laporte, R. L. , Gonzalez, K. A. , Toomey, R. B. , & Domenech Rodríguez, M. M. (2025). Latinx caregivers of LGBTQ people, cultural values, and mental health outcomes. Journal of Family Psychology, 39(4), 407–417. 10.1037/fam0001320 40193428

[jora70254-bib-0004] Abreu, R. L. , Gonzalez, K. A. , Rosario, C. C. , Pulice‐Farrow, L. , & Rodríguez, M. M. D. (2020). “Latinos have a stronger attachment to the family”: Latinx fathers' acceptance of their sexual minority children. Journal of GLBT Family Studies, 16(2), 192–210. 10.1080/1550428X.2019.1672232

[jora70254-bib-0005] Abreu, R. L. , Lefevor, G. T. , Barrita, A. , Gonzalez, K. A. , & Watson, R. J. (2023). Intersectional microaggressions, depressive symptoms, and the role of LGBTQ‐specific parental support in a sample of Latinx sexual and gender minority youth. Journal of Adolescence, 95, 584–595. 10.1002/jad.12139 36680329

[jora70254-bib-0006] Abreu, R. L. , Martin, J. , & Badio, K. (2023). Latinx LGBTQ people and their families: The role of Latinx cultural values, beliefs, and traditions. In J. M. Koch , E. Townsend , & R. Hubach (Eds.), Identity as resilience in minoritized communities: Strengths‐based approaches in research and practice (pp. 47–58). Springer.

[jora70254-bib-0007] Abreu, R. L. , Skidmore, S. , Barrita, A. , Sostre, J. P. , Lefevor, G. T. , & Watson, R. (2026). Substance use, parental and teacher support, and mental health outcomes among Latinx sexual and gender minority youth. Journal of Homosexuality, 73(4), 983–1004. 10.1080/00918369.2025.2496200 40353813

[jora70254-bib-0008] Abreu, R. L. , Sostre, J. P. , Martin, J. A. , Lockett, G. M. , & Vazquez, T. (2025). Latinx trans and gender diverse people's daily experiences of discrimination, violence, survival, and wellness: A writing approach. Journal of Counseling Psychology, 72(5), 433–445. 10.1037/cou0000813 40674024

[jora70254-bib-0009] Ancín‐Nicolás, R. A. , Pastor, Y. , López‐Sáez, M. Á. , & Platero, L. (2024). Protective factors in the LGBTIQ+ adolescent experience: A systematic review. Healthcare, 12(18), 1865. 10.3390/healthcare12181865 39337206 PMC11431086

[jora70254-bib-0010] Barrita, A. , Abreu, R. L. , Carbajal, I. , Parmenter, J. G. , & Watson, R. (2026). *Alcohol and native language*: Alcohol use as a coping strategy for intersectional microaggressions among sexual and gender minoritized Latinx youth. Journal of Psychoactive Drugs, 1–11. 10.1080/02791072.2026.2661582 PMC13215679 42026806

[jora70254-bib-0011] Barrita, A. , Abreu, R. L. , Parmenter, J. , & Watson, R. (2025). *Protective effects of online safety and parental acceptance for sexual and gender minority Latinx youth*: A quantitative analysis of cyberbullying, psychological distress, and coping with alcohol. LGBT Health, 12(7), 490–498. 10.1089/lgbt.2024.0396 40623828 PMC12629678

[jora70254-bib-0012] Barrita, A. , Abreu, R. L. , Zelaya, D. G. , Parmenter, J. G. , Williams, N. , & Watson, R. J. (2026). An intersectional approach for the effects of school bullying & cyberbullying on beliefs about the future among Latine LGBTQ+ youth in the United States. Journal of Research on Adolescence, 36, e70247. 10.1111/jora.70247 42572836 PMC13454855

[jora70254-bib-0013] Barrita, A. , Parmenter, J. , Abreu, R. L. , Sostre, J. P. , & Watson, R. (2025). Therapy and parental support for LGBTQ+ Latinx and black youth: A moderated mediation analysis of internalized stigma, school bullying, and psychological distress. Mental Health Science, 3(1), e70009. 10.1002/mhs2.70009

[jora70254-bib-0014] Beaton, D. E. , Bombardier, C. , Guillemin, F. , & Ferraz, M. B. (2000). Guidelines for the process of cross‐cultural adaptation of self‐report measures. Spine, 25(24), 3186–3191. 10.1097/00007632-200012150-00014 11124735

[jora70254-bib-0015] Brislin, R. W. (1970). Back‐translation for cross‐cultural research. Journal of Cross‐Cultural Psychology, 1, 185–216. 10.1177/135910457000100301

[jora70254-bib-0016] Brown, A. (2015). The unique challenges of surveying U.S. Latinos. Pew Research Center. Retrieved October 28, 2025, from https://www.pewresearch.org/social‐trends/2015/11/12/the‐unique‐challenges‐of‐surveying‐u‐s‐latinos‐2/

[jora70254-bib-0017] Cahill, K. M. , Updegraff, K. A. , Causadias, J. M. , & Korous, K. M. (2021). Familism values and adjustment among Hispanic/Latino individuals: A systematic review and meta‐analysis. Psychological Bulletin, 147(9), 947–985. 10.1037/bul0000336

[jora70254-bib-0018] Calzada, E. J. , Fernandez, Y. , & Cortes, D. E. (2010). Incorporating the cultural value of respeto into a framework of Latino parenting. Cultural Diversity & Ethnic Minority Psychology, 16(1), 77–86. 10.1037/a0016071 20099967 PMC4403003

[jora70254-bib-0019] Calzada, E. J. , Huang, K.‐Y. , Anicama, C. , Fernandez, Y. , & Brotman, L. M. (2012). Test of a cultural framework of parenting with Latino families of young children. Cultural Diversity & Ethnic Minority Psychology, 18(3), 285–296. 10.1037/a0028694 22686147 PMC4405111

[jora70254-bib-0020] DelFerro, J. , Whelihan, J. , Min, J. , Powell, M. , DiFiore, G. , Gzesh, A. , Jelinek, S. , Schwartz, K. T. G. , Davis, M. , Jones, J. D. , Fiks, A. G. , Jenssen, B. P. , & Wood, S. (2024). The role of family support in moderating mental health outcomes for LGBTQ+ youth in primary care. JAMA Pediatrics, 178(9), 914–922. 10.1001/jamapediatrics.2024.1956 38949835 PMC11217892

[jora70254-bib-0021] El Centro — University of Miami . (n.d.). CLaRO Demographics Form . https://elcentro.sonhs.miami.edu/research/measures‐library/claro/index.html

[jora70254-bib-0022] Estevez, R. , Singh, A. , Delgado‐Romero, E. , Pace, S. , Ozuna, C. , Hamilton, J. , Bockting, W. , & LeBlanc, A. (2025). “Seeing the balance in the two worlds in which I exist”: Latinx trans and nonbinary individuals' experiences of within‐culture gender minority stress and resilience. Journal of Counseling Psychology, 72(2), 158–171. 40095979 10.1037/cou0000787 PMC11948218

[jora70254-bib-0023] Fabrigar, L. R. , Wegener, D. T. , MacCallum, R. C. , & Strahan, E. J. (1999). Evaluating the use of exploratory factor analysis in psychological research. Psychological Methods, 4(3), 272–299. 10.1037/1082-989X.4.3.272

[jora70254-bib-0024] Galliher, R. V. , McLean, K. C. , & Syed, M. (2017). An integrated developmental model for studying identity content in context. Developmental Psychology, 53(11), 2011–2022. 10.1037/dev0000299 29094966

[jora70254-bib-0025] García Coll, C. , Crnic, K. , Lamberty, G. , Wasik, B. H. , Jenkins, R. , Garcia, H. V. , & McAdoo, H. P. (1996). An integrative model for the study of developmental competencies in minority children. Child Development, 67(5), 1891–1914. 10.1111/j.1467-8624.1996.tb01834.x 9022222

[jora70254-bib-0026] Gianola, M. , Llabre, M. M. , & Losin, E. A. R. (2025). Language dominance and cultural identity predict variation in self‐reported personality in English and Spanish among Hispanic/Latino bilingual adults. Journal of Personality Assessment. Advance online publication. 10.1080/00223891.2024.2416412 PMC11981866 39514799

[jora70254-bib-0027] Gonzalez, D. , Nelson, T. , & Smedley, B. (2025). One in four Spanish‐speaking Hispanic adults have difficulty finding language‐concordant health care providers. Urban Institute. https://www.urban.org/sites/default/files/2025‐05/One‐in‐Four‐Spanish‐Speaking‐Hispanic‐Adults‐Have‐Difficulty‐Finding‐Language‐Concordant‐Health‐Care‐Providers.pdf

[jora70254-bib-0028] Halgunseth, L. C. , Ispa, J. M. , & Rudy, D. (2006). Parental control in Latino families: An integrated review of the literature. Child Development, 77(5), 1282–1297. 10.1111/j.1467-8624.2006.00934.x 16999798

[jora70254-bib-0029] Hayton, J. C. , Allen, D. G. , & Scarpello, V. (2004). Factor retention decisions in exploratory factor analysis: A tutorial on parallel analysis. Organizational Research Methods, 7(2), 191–205. 10.1177/1094428104263675

[jora70254-bib-0030] Hooper, D. , Coughlan, J. , & Mullen, M. R. (2008). Structural equation modelling: Guidelines for determining model fit. Electronic Journal of Business Research Methods, 6(1). https://academic‐publishing.org/index.php/ejbrm/article/view/1224

[jora70254-bib-0031] Knight, G. P. , Carlo, G. , Streit, C. , & White, R. M. (2017). A model of maternal and paternal ethnic socialization of Mexican‐American adolescents' self‐views. Child Development, 88(6), 1885–1896. 10.1111/cdev.12939 28857150 PMC5675779

[jora70254-bib-0032] Martin, J. , Barrita, A. , & Abreu, R. L. (2025). Social justice and race moderated parental acceptance and mental health in BIPOC caregivers of LGBTQ youth: Implications for family therapy interventions. Translational Issues in Psychological Science, 11(4), 467–476. 10.1037/tps0000462

[jora70254-bib-0033] Moos, R. H. , & Moos, B. S. (1994). Family environment scale manual. Consulting Press.

[jora70254-bib-0034] Morrison, M. A. , & Morrison, T. G. (2002). Development and validation of a scale measuring modern prejudice toward gay men and lesbian women. Journal of Homosexuality, 43(2), 15–37. 10.1300/J082v43n02_02 12739696

[jora70254-bib-0035] Nair, R. L. , White, R. M. B. , Knight, G. P. , & Roosa, M. W. (2009). Cross‐language measurement equivalence of parenting measures for use with Mexican American populations. Journal of Family Psychology, 23(5), 680–689. 19803604 10.1037/a0016142 PMC2760044

[jora70254-bib-0036] Parmenter, J. G. , Lê, D. , Barrita, A. , Muskrat, T. , Easton, A. , Staton, D. , & Trenary, J. (2026). “I think I take on the whole system”: Experiences of intersectional proximal minority stressors among queer, transgender, and nonbinary people of color. Cultural Diversity & Ethnic Minority Psychology. Advance online publication. 10.1037/cdp0000789 41525370

[jora70254-bib-0037] Parra‐Cardona, J. R. , Domenech Rodríguez, M. , Forgatch, M. S. , Sullivan, C. , Bybee, D. , Tams, L. , Holtrop, K. , Escobar‐Chew, A. R. , Dates, B. , & Bernal, G. (2012). Culturally adapting an evidence‐based parenting intervention for Latinos: Preliminary implications for family therapy practice and research. Family Process, 51(1), 56–72. 10.1111/j.1545-5300.2012.01386.x 22428711 PMC3313069

[jora70254-bib-0038] Peña, E. D. (2007). Lost in translation: Methodological considerations in cross‐cultural research. Child Development, 78(4), 1255–1264. 10.1111/j.1467-8624.2007.01064.x 17650137

[jora70254-bib-0039] Pew Research Center . (2022). A brief statistical portrait of U.S. Hispanics. C. Funk & M. H. Lopez. https://www.pewresearch.org/science/2022/06/14/a‐brief‐statistical‐portrait‐of‐u‐s‐hispanics/

[jora70254-bib-0040] Ramos, G. , Lorenzo, N. E. , Garcia, D. , & Bagner, D. M. (2023). Skill change among Latinx families in a behavioral parenting intervention: The interactive effect of caregiver language preference and acculturation. Journal of Latinx Psychology, 11(3), 175–188. 10.1037/lat0000226 37810445 PMC10557956

[jora70254-bib-0041] Rohner, R. P. (2021). Introduction to Interpersonal Acceptance‐Rejection Theory (IPARTheory) and Evidence. Online Readings in Psychology and Culture, 6(1). 10.9707/2307-0919.1055

[jora70254-bib-0042] Rohner, R. P. , & Ali, S. (2020). Parental Acceptance‐Rejection Questionnaire (PARQ). In V. Zeigler‐Hill & T. K. Shackelford (Eds.), Encyclopedia of personality and individual differences (pp. 3425–3427). Springer. 10.1007/978-3-319-24612-3_56

[jora70254-bib-0043] Romero, A. J. , Robinson, T. N. , Haydel, K. F. , Mendoza, F. , & Killen, J. D. (2004). Associations among familism, language preference, and education in Mexican‐American mothers and their children. Journal of Developmental and Behavioral Pediatrics, 25(1), 34–40. 10.1097/00004703-200402000-00006 14767354

[jora70254-bib-0044] Rosseel, Y. (2012). lavaan: An R package for structural equation modeling. Journal of Statistical Software, 48(1), 1–36.

[jora70254-bib-0045] Russell, S. T. , & Fish, J. N. (2019). Sexual minority youth, social change, and health: A developmental collision. Research in Human Development, 16(1), 5–20. 10.1080/15427609.2018.1537772 31602178 PMC6786797

[jora70254-bib-0046] Ryan, C. , Huebner, D. , Diaz, R. M. , & Sanchez, J. (2009). Family rejection as a predictor of negative health outcomes in white and Latino lesbian, gay, and bisexual young adults. Pediatrics, 123(1), 346–352. 10.1542/peds.2007-3524 19117902

[jora70254-bib-0047] Ryan, C. , Russell, S. T. , Huebner, D. , Diaz, R. , & Sanchez, J. (2010). Family acceptance in adolescence and the health of LGBT young adults. Journal of Child and Adolescent Psychiatric Nursing, 23(4), 205–213. 10.1111/j.1744-6171.2010.00246.x 21073595

[jora70254-bib-0048] Sawyer, S. M. , Azzopardi, P. S. , Wickremaratne, D. , & Patton, G. C. (2018). The age of adolescence. The Lancet Child & Adolescent Health, 2(3), 223–228.30169257

[jora70254-bib-0049] Sousa, V. D. , & Rojjanasrirat, W. (2011). Translation, adaptation and validation of instruments or scales for use in cross‐cultural health care research: A clear and user‐friendly guideline. Journal of Evaluation in Clinical Practice, 17(2), 268–274. 10.1111/j.1365-2753.2010.01434.x 20874835

[jora70254-bib-0050] Tabachnick, B. , & Fidell, L. S. (2001). Using multivariate statistics (4th ed.). Allyn & Bacon.

[jora70254-bib-0051] Van de Vijver, F. J. R. , & Leung, K. (1997). Methods and data analysis of comparative research. In J. W. Berry , Y. H. Poortinga , & J. Pandey (Eds.), Handbook of cross‐cultural psychology (Vol. 1, pp. 257–300). Allyn & Bacon.

[jora70254-bib-0053] Vazquez, T. , Costa, R. , Barrita, A. , & Abreu, R. L. (2025). Perceived election safety, resilience, caregiver acceptance and psychological well‐being among LGBTQ+ young adults: A parallel mediation model. Sexuality Research & Social Policy, 1–13. 10.1007/s13178-025-01244-y

[jora70254-bib-0052] Vázquez, A. L. , Domenech Rodríguez, M. M. , Cadenas, G. A. , Barrett, T. S. , & Navarro Flores, C. M. (2024). Barriers to accessing telepsychology services questionnaire: Structure and language‐based performance in a sample of Latinx caregivers. Psychological Services, 21(1), 50–64. 10.1037/ser0000804 37856391

[jora70254-bib-0054] Wenz, A. , Al Baghal, T. , & Gaia, A. (2021). Language proficiency among respondents: Implications for data quality in a longitudinal face‐to‐face survey. Journal of Survey Statistics and Methodology, 9(1), 73–93. 10.1093/jssam/smz045

[jora70254-bib-0055] Worthington, R. L. , & Whittaker, T. A. (2006). Scale development research: A content analysis and recommendations for best practices. The Counseling Psychologist, 34(6), 806–838. 10.1177/0011000006288127

[jora70254-bib-0056] Young, S. R. , Freer, C. , Gefen, N. , Gonzalez, I. , Kemps, R. , Partanen, M. , & Colbert, A. (2024). Cross‐cultural collaborative translation/adaptation of assessments via international working groups: A case study with the cognitive and linguistic scale. International Perspectives in Psychology, 13(2), 79–87. 10.1027/2157-3891/a000094

