# Supplementary material for: Bridging language and culture: The LGBTQ Caregiver Acceptance Scale's Spanish (LCAS‐S) translation and validation among Latinx caregivers
Source: J Res Adolesc. 2026 Aug 19;36(3):e70254. doi: 10.1111/jora.70254 (PMC13487645; doi:10.1111/jora.70254)
Supplement: Supplementary file 1 [file JORA-36-0-s001.docx]

**Supplemental Table 1**

*LGBTQ+ Person Demographics (Reported by Caregiver;* N *= 256)*

| Demographic Variable | *N* (%) |
| --- | --- |
| *Sexual Identity of Child/Family Member^*^* |  |
| Lesbian | 72 (28.1) |
| Gay | 93 (36.3) |
| Bisexual | 76 (29.7) |
| Queer | 12 (4.7) |
| Other (e.g., pansexual, fluid) | 26 (10.2) |
| Heterosexual | 15 (5.9) |
| *Gender Identity of LGBTQ+ Child/Family Member*  Man | 97 (37.9) |
| Woman | 122 (47.7) |
| Non-binary | 37 (14.5) |

**Supplemental Table 2**

*LCAS versus LCAS-S item comparison for placement or removal*

| Items | Kept or Removed | LCAS-S Factor | Reason for removal |
| --- | --- | --- | --- |
| LCAS Factor 1: Outness |  |  |  |
| It was fine that my LGBTQ child revealed their identity to family friends. | Kept | Outness | -- |
| I am worried that my family would reject me because I have an LGBTQ child. | Kept | Fear of Family Rejection | -- |
| It was fine that my LGBTQ child revealed their identity to the members of my community (for example, priests, pastors, members of my church, neighbors). | Kept | Outness | -- |
| It was fine that my LGBTQ child revealed their identity to other family members. | Kept | Outness | -- |
| I am worried about what my family will say if they find out that my child is LGBTQ. | Kept | Fear of Family Rejection | -- |
| I consider that it is disrespectful for my child to show any symbol that others could associate with being LGBTQ (for example, pride flag). | Removed | -- | Loaded as factor 7***** but lacked conceptual cohesion |
| It is fine for my LGBTQ child to attend family activities. | Kept | Affirming LGBTQ child’s place in family | -- |
| I am worried that my family would reject my LGBTQ child. | Kept | Fear of Family Rejection | -- |
| Accepting my LGBTQ child is a way of keeping the family united. | Kept | Affirming LGBTQ child’s place in family | -- |
| It is fine for my child’s romantic partner to get involved in family decisions | Removed | -- | Minimum loading <.40 (.287) item may reflect future-oriented parenting beliefs less central to immediate adolescent caregiving experiences |
| LCAS Factor 2: Caregiver Acceptance |  |  |  |
| I feel that I have to choose between my LGBTQ child and the rest of my family. | Kept | Concealment and Respeto | -- |
| It is difficult to accept my LGBTQ child. | Removed | -- | Minimum cross-loading <.30 (.086) item may reflect future-oriented parenting beliefs less central to immediate adolescent caregiving experiences |
| I am worried that my family would ask me to choose between my LGBTQ child and the rest of my family. | Kept | Fear of Family Rejection | -- |
| It is difficult to accept my LGBTQ child’s romantic partner. | Removed | -- | Loaded as factor 7***** but lacked conceptual cohesion |
| I am worried that my child’s sexual orientation could cause conflicts in the family. | Kept | Fear of Family Rejection | -- |
| I have sought support from other family members to help me accept my LGBTQ child. | Removed | -- | Minimum cross-loading <.30 (.122) item may reflect future-oriented parenting beliefs less central to immediate adolescent caregiving experiences |
| LCAS Factor 3: Concealment |  |  |  |
| I wish my child would not reveal their sexual orientation or gender identity to family friends. | Kept | Concealment and Respeto | -- |
| Taking into account the opinions of the family is more important than being openly LGBTQ. | Kept | Concealment and Respeto | -- |
| I wish my child would keep their LGBTQ identity a secret to protect the family. | Kept | Concealment and Respeto | -- |
| My child must keep their LGBTQ identity a secret in order to be considered part of the family. | Kept | Concealment and Respeto | -- |
| Protecting the image of the family is more important than allowing my child to express their sexual orientation or gender identity. | Kept | Concealment and Respeto | -- |
| It would be easier for some members of my family to accept my LGBTQ child if they weren’t openly LGBTQ. | Removed | -- | Loaded as factor 7***** but lacked conceptual cohesion |
| LCAS Factor 4: Respeto |  |  |  |
| I have asked my LGBTQ child not to act “gay” in front of members of my community (for example, priests, pastors, members of my church, neighbors). | Kept | Public Opinion | -- |
| It is disrespectful for my child to speak openly about their romantic relationship. | Removed | -- | Minimum cross-loading <.30 (.149) item may reflect future-oriented parenting beliefs less central to immediate adolescent caregiving experiences |
| Being openly LGBTQ is disrespectful to the family. | Kept | Concealment and Respeto | -- |
| It is disrespectful for my child to express their LGBTQ identity. | Remove | -- | Minimum loading <.40 (.185) item may reflect future-oriented parenting beliefs less central to immediate adolescent caregiving experiences |
| Acting "gay” is disrespectful. | Kept | Concealment and Respeto | -- |
| I would prefer not to reveal my child's sexual orientation or gender identity to members of my community (for example, priests, pastors, members of my church, neighbors) that would not support them. | Kept | Concealment and Respeto | -- |
| I have asked my priest/pastor/spiritual leader about how to deal with my LGBTQ child. | Kept | Public Opinion | -- |
| I have asked other people whose opinion is important to me for advice about how to deal with my LGBTQ child. | Kept | Public Opinion | -- |
| I thought about the feelings of family members before speaking openly about my LGBTQ child. | Removed | -- | Loaded as factor 7***** but lacked conceptual cohesion |
| LCAS Factor 5: Attitudes Towards Queer Parenting |  |  |  |
| It is not appropriate for my LGBTQ child and their romantic partner to raise children. | Removed | -- | Minimum cross-loading <.30 (.093) |
| It is not appropriate for my LGBTQ child to raise children | Removed | -- | Minimum loading <.40 (.107) item may reflect future-oriented parenting beliefs less central to immediate adolescent caregiving experiences |
| I would consider my LGBTQ child’s children to be my grandkids. | Kept | Affirming LGBTQ child’s place in family | -- |
| It is fine for my LGBTQ child and their romantic partner to have children. | Removed | -- | Minimum loading <.40 (.242) item may reflect future-oriented parenting beliefs less central to immediate adolescent caregiving experiences |
| LCAS Factor 6: Supportive Actions |  |  |  |
| I have invited my child’s romantic partner(s) to participate in family activities. | Kept | Supportive Actions | -- |
| I have asked family members to support my LGBTQ child. | Removed | -- | Loaded as factor 7***** but lacked conceptual cohesion |
| I have invited my child's romantic partner(s) to participate in family matters. | Kept | Supportive Actions | -- |
| I would participate in LGBTQ Pride events to show support to my LGBTQ child. | Kept | Supportive Actions | -- |
| I chose my LGBTQ child over my family. | Kept | Supportive Actions | -- |

***Note. Items loading onto Factor 7 reflected heterogeneous themes related to restriction, family discomfort, and anticipated social judgment; however, the factor lacked sufficient conceptual coherence and theoretical distinctiveness to justify retention as an independent dimension of LGBTQ+-related caregiver acceptance.**
